# Supplementary material for: Asynchronous magnetic resonance elastography: Shear wave speed reconstruction using noise correlation of incoherent waves
Source: Magn Reson Med. 2022 Oct 27;89(3):990–1001. doi: 10.1002/mrm.29502 (PMC9792433; doi:10.1002/mrm.29502)
Supplement: Supplementary file 1 — DATA S1 MATLAB code used to generate the numerical simulations of Figure 1 [file MRM-89-990-s002.zip › k-Wave/helpfiles/spect.html]

spect :: Functions (k-Wave)


# spect

Compute the single sided amplitude and phase spectrums.

## Syntax

```
func_as = spect(func, Fs)
func_as = spect(func, Fs, ...)
[f, func_as] = spect(func, Fs)
[f, func_as] = spect(func, Fs, ...)
[f, func_as, func_ps] = spect(func, Fs)
[f, func_as, func_ps] = spect(func, Fs, ...)
```

## Description

`spect` computes the single-sided amplitude and phase spectrums of a 1, 2, or 3 dimensional input signal. If the optional input `'Dim'` is not specified, the spectrum is computed along the first non-singleton dimension. For example, running the commands

```
% define signal
Fs = 10e6;              % [Hz]
dt = 1 / Fs;            % [s]
t = 0:dt:(1000 * dt);   % [s]
signal = 10 * sin(0.5e6 * 2 * pi * t) + 5 * sin(1e6 * 2 * pi * t) + 2 * sin(2.5e6 * 2 * pi * t);

% calculate spectrum
spect(signal, Fs, 'Plot', [true, false]);
```

will produce the following amplitude spectrum.

## Inputs

|  |  |
| --- | --- |
| `func` | signal to analyse |
| `Fs` | sampling frequency [Hz] |

## Optional Inputs

Optional 'string', value pairs that may be used to modify the default computational settings.

| Input | Valid Settings | Default | Description |
| --- | --- | --- | --- |
| `'Dim'` | *(integer scalar)* | *first non-singleton dimension* | Dimension over which the spectrum is computed. |
| `'FFTLength'` | *(integer scalar)* | *signal length* | Length of the FFT. If the set length is larger than the signal length, the signal is zero padded. If the set length is smaller than the signal length, the default value is used instead. |
| `'PowerTwo'` | *(Boolean scalar)* | `false` | Boolean controlling whether the FFT length is forced to be the next highest power of 2. |
| `'Plot'` | *(Boolean scalar or two element vector)* | `false` | Boolean controlling whether the amplitude and phase spectrums are plotted. Can be given as a two element array to control the display of the amplitude and phase spectrums, respectively. |
| `'Unwrap'` | *(Boolean scalar)* | `false` | Boolean controlling whether the phase spectrum is unwrapped. |
| `'Window'` | *(string)* | `'Rectangular'` | Parameter string controlling the window type used to filter the signal before the FFT is taken. Any valid input types for `getWin` may be used. |

## Outputs

|  |  |
| --- | --- |
| `f` | frequency array |
| `func_as` | single sided amplitude spectrum |
| `func_ps` | single sided phase spectrum |

## Examples

- Defining A Gaussian Sensor Frequency Response
- The Doppler Effect
- Filtering A Delta Function Input Signal
- Image Reconstruction With Compensation For Acoustic Absorption
- Source Smoothing
- Filtering A Delta Function Input Signal
- Modelling Power Law Absorption

## See Also

`smooth`, `filterTimeSeries`
